# Drug Holiday of Interferon Beta 1b in Multiple Sclerosis: A Pilot, Randomized, Single Blind Study of Non-inferiority

**DOI:** 10.3389/fneur.2019.00695

**Published:** 2019-07-16

**Authors:** Silvia Romano, Michela Ferraldeschi, Francesca Bagnato, Rosella Mechelli, Emanuele Morena, Marzia Caldano, Maria Chiara Buscarinu, Arianna Fornasiero, Marco Frontoni, Viviana Nociti, Massimiliano Mirabella, Flavia Mayer, Antonio Bertolotto, Carlo Pozzilli, Nicola Vanacore, Marco Salvetti, Giovanni Ristori

**Affiliations:** ^1^Neurosciences, Mental Health, and Sensory Organs (NESMOS) Department, Center for Experimental Neurological Therapies, S. Andrea Hospital-site, “Sapienza” University of Rome, Rome, Italy; ^2^Department of Neurology and Psychiatry, “Sapienza” University of Rome, Rome, Italy; ^3^Neuroimmunology Division, Department of Neurology, Neuroimaging Unit, Vanderbilt University Medical Centre, Nashville, TN, United States; ^4^Neurologia – Centro Riferimento Regionale Sclerosi Multipla and Neuroscience Institute Cavalieri Ottolenghi, San Luigi Hospital, Turin, Italy; ^5^D.A.I. Neurosciences and Mental Health, “Sapienza” University of Rome, Rome, Italy; ^6^Università Cattolica, Fondazione Policlinico Universitario “A. Gemelli, ” Rome, Italy; ^7^Don Carlo Gnocchi Foundation Onlus, Milan, Italy; ^8^National Centre of Epidemiology, National Institute of Health, Rome, Italy; ^9^IRCCS Istituto Neurologico Mediterraneo (INM) Neuromed, Pozzilli, Italy

**Keywords:** relapsing-remitting multiple sclerosis, interferon beta 1b, non-inferiority, cyclic withdrawal, contrast-enhanced lesions, black holes

## Abstract

**Introduction:** To compare a schedule with cyclic withdrawal (CW) of interferon beta (IFN-b) 1b, respect to the full regimen (FR), in relapsing-remitting MS (RR-MS).

**Methods:** Participants were randomly assigned to CW or FR schedule and monthly monitored with brain MRI scans for 12 months (three of run-in and 9 of treatment). CW schedule included drug withdrawal for 1 month after two of treatment for a total of three quarters over the 9-month treatment period. The assessing neurologist and the expert neuroradiologists were blind. After the blind phase of the study all participants took their indicated disease modifying therapies in a prospectively planned, open-label extension phase (up to 120 months).

**Results:** Of 60 randomized subjects 56 (29 in FR and 27 in CW group) completed the single-blind phase: the two groups were comparable, except for a non-significant difference in the number of contrast-enhanced lesions (CEL) at the end of run-in. The two-sided 90% CI for the ratio between median number of cumulative CEL was 0.29–1.07, allowing to significantly reject the null hypothesis of a ratio ≥1.2 and to meet the primary end-point of non-inferiority (the threshold and the ratio between median were chosen according to the non-normal distribution of the data). The differences (CW vs. FR) were also non-significant for secondary end points: mean cumulative number of T2-weighted new and enlarging lesions (3.48 ± 5.34 vs. 3.86 ± 6.76); mean number and volume (cm^3^) of black holes (1.24 ± 1.61 vs. 2.71 ± 4.56; 489.11 ± 1488.12 vs. 204.48 ± 396.98); number of patients with at least an active scan (21 vs. 22); mean relapse rate (0.52 ± 0.89 vs. 0.34 ± 0.66), relapse risk ratio adjusted for baseline variables (2.15 [0.64–7.18]), EDSS score (1.0 [1–1.56] vs. 1.5 [1–1.78]), proportion of patients with antibodies anti-IFN (5 [21%] vs. 8 [36%]). Fifty-four patients (27 for each study arm) completed the open-label phase. The annualized RR, EDSS, proportion of patients shifting to progressive disease and hazard ratio of shifting, adjusting for baseline covariates, were comparable between the two study groups.

**Conclusions:** A calendar with CW was non-inferior than FR at the beginning of IFN-b therapy, and may not affect the long-term outcome.

**Clinical Trial Registration:** www.ClinicalTrials.gov, identifier: NCT00270816

## Introduction

Relapsing remitting multiple sclerosis (RR-MS) has become manageable thanks to a wide range of new treatments (1). However, these therapies last for decades and raise issues of safety, reduction in life quality, treatment adherence, and healthcare expenditure (2).

Furthermore, inflammation, neurodegeneration, and metabolic modifications co-exist and all need therapeutic intervention in MS; however, none of the approved disease modifying therapies (DMT) has been proven to tackle them all. The increasingly shared view is that combining therapies with different mechanisms of action may target the complex pathophysiology of the disease (3–5).

Hence, given the characteristics of available treatments, and within the perspective of facilitating the development of combination therapies, new regimens that reduce the treatment burden represent an unmet need in MS.

Work in our group using serial transcriptome analysis in peripheral blood mononuclear cells (PBMC) of patients treated with interferon beta (IFN-b) showed that gene expression changes are more pronounced during the 1st weeks of treatment, with a clear tendency to return to baseline levels 2–3 months after treatment initiation (6). This result provided the rationale for the design of a treatment regimen that includes cyclic withdrawals (CW) of treatment with IFN-b 1b. This schedule may in principle maintain the biological impact that is gradually lost due to the homeostatic response, be less cumbersome for patients and less expensive for the healthcare system. We therefore tested the hypothesis of non-inferiority of a schedule based on CW of IFN-b 1b compared to the canonical, full regimen (FR) in persons with RR-MS.

## Patients and Methods

### Standard Protocol Approvals, Registrations, and Patient Consents

The present single-blind, phase-IV, randomized clinical study was performed at the Center for Experimental Neurological Therapies, Ospedale S. Andrea-site, “Sapienza” University of Rome, and Università Cattolica, Fondazione Policlinico Universitario “A. Gemelli,” Rome, Italy. The study was registered at the ClinicalTrial.gov (NCT00270816). The trial was conducted according to Good Clinical Practice guidelines and the Declaration of Helsinki. The protocol was approved by the Ethics Committee of Ospedale S. Andrea, “Sapienza” University of Rome and each patient provided written informed consent.

### Patients

Patients with RR-MS (7) were consecutively screened and enrolled between November 2006 and November 2008. The following were considered as inclusion criteria: age between 18 and 50 years (inclusive); no steroid treatment in the 2 months prior to the study and no previous exposure to any DMT. Exclusion criteria were: systemic diseases; pregnancy, inability to agree on the use of contraception for women of childbearing potential and breast-feeding, inability to undergo magnetic resonance imaging (MRI).

### Protocol

The study design included a 3-month run-in period (months 1–3) and a 9-month treatment period (months 4–12). At the end of the run-in, patients were 1:1 randomized to receive either IFN-b 1b every other day consecutively (FR arm) or to CW arm (drug withdrawal for 1 month after two of treatment for a total of three quarters over the 9-month treatment period). Patients of the CW arm stopped IFN beta-1b 3 times, at months 6, 9, and 12. A list of randomization numbers and corresponding treatment numbers was computer-generated before the start of the study. A 2-physician-treating and assessing-model was used: the treating physician supervised drug administration, evaluated adverse events, and safety; the assessing physician evaluated outcome measures and was blind.

A prospectively planned, extension of the trial was conducted up to 120 months, during which the patients remained under the FR standard schedule of IFN-b 1b, or were shifted to the DMT that their neurologist in charge considered indicated.

### Procedures

At first visit each patient underwent a clinical examination to rate disability with the Expanded Disability Status Score (EDSS) scale (8), performed routine blood tests, any required test to rule out MS mimickers, electrocardiogram, and brain MRI scan. Thereafter, each patient underwent monthly clinic visit and MRIs for the first 12 months, and every 6-month clinical with or without imaging assessment till month 120.

Clinical relapses were defined as the appearance of new symptoms or worsening of previous symptoms/signs associated with changes in the neurological examination lasting longer than 24 h in the absence of fever, infections or any other acute process that could be responsible for neurological worsening. Adverse events were defined as any untoward medical occurrence regardless of its causal relationship to the study treatment. The severity of the adverse events was graded as: mild (minimal or no required treatment and no interference with the patient's daily activities); moderate (low level of inconvenience or concern; may require therapeutic measures and cause some interference with functioning); severe (interruption of a patient's usual daily activities and requirement of systemic drug therapy or other treatment; usually incapacitating); life-threatening (immediate risk of death).

At the above specified time points all participants were imaged with gadolinium (Gd)-enhanced MRI of brain to calculate the number of contrast enhancing lesions (CELs) and the other outcome measures (see below). MRI was performed using a 1.5-T magnet (Philips Gyroscan NT 1.5). The magnet underwent several upgrades during the study period and care was taken that none of these upgrades affected the sequences in use. T2-weighted (T2W) spin-echo (SE) (TR = 2,000 ms; TE = 20/90 ms), fast fluid-attenuated inversion-recovery (FLAIR) (TR = 6,000 ms; TE = 150 ms) and T1-weighted (T1W) SE (TR = 550 ms; TE = 12 ms) sequences were acquired in the axial plane with 5-mm contiguous slices, and a field of view (FOV) = 240 mm, matrix = 256 X256. The hardcopy was analyzed by two experienced neuroradiologists working in pairs (when there was a disagreement a third senior neuroradiologist reviewed the images and a final consensus was reached). They were blind and detected the number of Gd**-**enhancing lesions, T2**-**hyperintense lesions, and T1-hypointense lesions. The number of chronic black holes (BH) on T1W MRI was assessed according to Bagnato et al. (9).

Neutralizing antibodies (NAB) status was evaluated with a bioassay based on the cytopathic effect (CPE) of encephalomyocarditis virus (EMC) on human lung carcinoma cells (A549) as previously described (10, 11). The assay was performed prior to study entry and at the end of the 9-month treatment phase. The neutralization titer of serum samples was calculated according to Kawade's formula (12), and expressed in 10-fold reduction unit (TRU) (13). A level of >20 TRU was considered as the threshold for positivity.

### Outcomes Measures

Group (CW vs. FR) differences in the cumulative number of CELs over the first 9-month period was defined as the primary end point. Secondary end points included: group differences in cumulative number of new and enlarging T2-weighted lesions, number of patients with at least an active scan, relapse rate, number, and volume of BH, EDSS score, and proportion of NAB + patients. The outcome measures of the open label phase were the following: annualized relapses rate (ARR), EDSS, proportion of patients shifting to progressive MS [defined according to Lublin et al. (14) and Lublin et al. (15)].

### Statistical Analysis

Group differences at study entry were assessed using a *t*-test for continuous variables and a chi-square test for categorical variables. For continuous variables, median and Interquartile Range (IQR) were also calculated. When the variable distribution was non-normal according to Shapiro-Wilk test, the two groups were compared performing a non-parametric K-sample test on the equality of medians.

At the end of the single-blind phase of the trial, mean, standard deviation, median, IQR, and the range values were calculated for the primary and secondary endpoints, separately for CW and FR patients. For the primary outcome, we established to compare the number of CELs between the two regimens thorough the mean difference or the ratio of medians according to the normal or non-normal distribution of the data. In case of normality distribution of the data, we considered 8.1 as the mean number of CELs observed in the FR regimen in a previous work of our group (observed by Pozzilli et al. in a period of 6 months) (16) and operatively set a difference of 1.5 cumulative CELs for non-inferiority of the CW vs. the FR schedule, and 2 cumulative CELs of standard deviation. In this scenario, we rejected the null hypothesis with a significance level of 0.05 if the upper limit of the two-sided 90% confidence interval (CI) of the difference between the means of the 2 regimens was < 1.5 CEL (17, 18). To compute a one-sided testing procedure for the difference of the means, with an alpha error = 5% and a study power = 0.80, a sample size of 46 patients was required. Thirty patients per group were recruited to account for a drop-out rate of 20%. Power analyses were performed using StudySize software (version 2.0).

In case of non-normal distribution of the data, we operationally considered a non-inferiority threshold for the ratio of median of 1.2, assuming a standard deviation on elog Scale of 0.3. In this case, recruiting 30 patients per group allow to get a power of 0.90 for a one sided 95% CI. The null hypothesis was that the ratio of the median of the CW group on the median of the FR group was ≥ 1.2 and therefore the non-inferiority was demonstrated if the upper limit of the two sided 90% CI for the ratio of medians was lower than 1.2. Normality was tested using the Shapiro-Wilk test.

For the secondary endpoints, we calculated the relapse risk ratio (RR) using a Poisson model adjusted for the baseline variables (gender, age, disease duration, relapse number before study entry, EDSS, and MRI metrics), and performed a non-parametric K-sample test on the equality of medians for the EDSS score and the MRI metrics, that showed a non-normal distribution. During the single-blind phase of the trial an intention to treat analysis was performed adopting a LOCF (last observation carried forward) method.

During the open-label extension of the trial, we compared the annual relapse rate and the EDSS score between CW and FR groups performing a non-parametric K-sample test on the equality of medians, because of the non-normal distribution of the above variables. The proportion of patients who shifted to a progressive disease was compared using a chi-square test. Individual follow-up started with the beginning of the open label phase and finished at the end of the follow-up period (120 months), death, loss at follow-up, or shifting to a progressive disease.

## Results

### Patients Disposition and Characteristics

**Figure 1:**
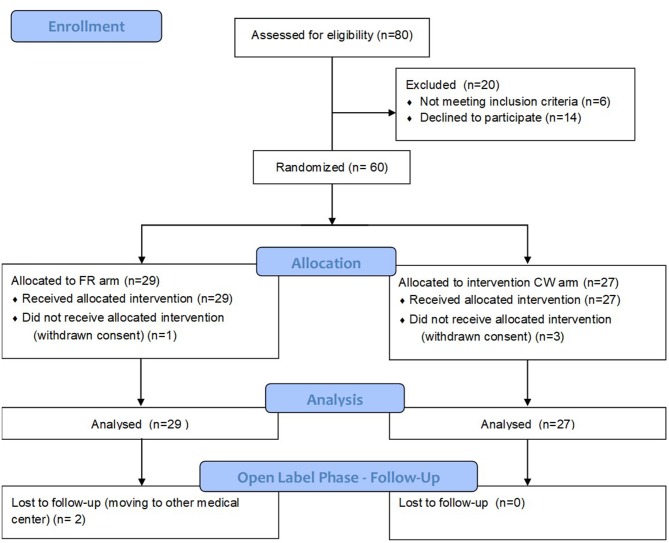
CONSORT flow diagram.

Figure 1 depicts eligibility assessment, patients screening, and inclusion. Table 1 reports baseline demographic and clinical characteristics of patients, as well as MRI metrics after the run in period. All the variables, including those showing a non-normal distribution, were balanced between the two groups.

**Table 1:** Baseline demographic and clinical metrics, along with MRI outcome measures after run-in.

**Patient characteristic**	**FR (*n* = 29)**	**CW (*n* = 27)**	***P*-value**
**AGE, YEARS**			
Mean ± SD	37.07 ± 7.25	35.70 ± 8.03	0.507^a^
Median (IQR)	39 (33.50–40.50)	37 (30–40)	
**SEX**			
M/F	12/17	10/17	0.789^b^
**DISEASE DURATION, MONTHS**			
Mean ± SD	48.45 ± 61.43	82.89 ± 91.54	0.130^c^
Median (IQR)	15 (6–65)	48 (10–156)	
**RELAPSE NUMBER^d^**			
Mean ± SD	2.25 ± 1.29	2.38 ± 1.33	0.714^a^
Median (IQR)	2 (1–3)	2.31 (1–3)	
**EDSS SCORE**			
Mean ± SD	1.49 ± 1.28	1.25 ± 0.73	0.941^c^
Median (IQR)	1.5 (1–1.75)	1 (1–1.5)	
**CUMULATIVE NUMBER OF GD-ENHANCING LESIONS AT MRI**			
Mean ± SD	5.66 ± 11.58	2.81 ± 4.05	0.486^c^
Median (IQR)	1 (0–3)	0 (0–4)	
**CUMULATIVE NUMBER OF NEW AND ENLARGING T2-WEIGHTED LESIONS AT MRI**			
Mean ± SD	2.93 ± 5.61	1.74 ± 3.94	0.483^c^
Median (IQR)	1 (0–2)	0 (0–2)	
**NUMBER OF PATIENTS WITH AT LEAST AN ACTIVE SCAN DURING RUN-IN**			
*N*	20	18	0.854^b^
	**(*****n*** **=** **24)**^e^	**(*****n*** **=** **25)^e^**	
**BLACK HOLES NUMBER ^f^**			
Mean ± SD	2.58 ± 4.46	1.20 ± 1.80	0.879^c^
Median (IQR)	0 (0–4)	0 (0–2)	
Range	0–18	0–7	
**BLACK HOLES–OVERALL VOLUME^f^** **(cm**^**3**^**)**			
Mean ± SD	222.06 ± 442.83	586.92 ± 1523.14	0.831^c^
Median (IQR)	0 (0–208.62)	0 (0–371.93)	
Range	0–1659.39	0–6673.33	

a *t-test for continuous variables*.

b *Chi-square test for categorical variables*.

c *Non-parametric Wilcoxon rank-sum test (for variables with non-normal distribution)*.

d *Before study entry*.

e *The baseline demographic and clinical characteristics of this subgroup were not different from those of the 7 patients lost for this analysis*.

f *After run-in*.

### Outcome Measures

**Figure 2:**
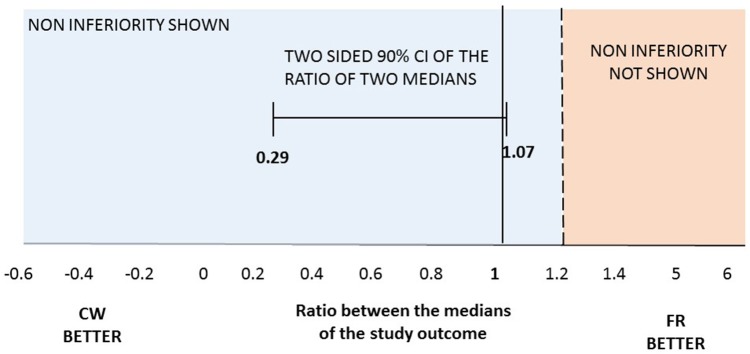
Two-sided 90%CI for the ratio between median number of cumulative Gd-enhancing lesions at MRI observed in the two regimens. Maximum ratio of 1.2 lesions was set for the one-tailed test with a significance level of 0.05. At month 12 median of the cumulative number of Gd-enhancing lesions at MRI was 1 (0–9) in CW vs. 3 (0–10) in FR patients.

At the end of the trial, we verified that the normality was not satisfied for the primary outcome. Therefore, we tested the non-inferiority comparing the medians of the two groups. The median number of cumulative Gd-enhancing lesions at MRI was comparable between the two study arms, meeting the primary end-point: median 3 and IQR (0–10) for FR vs. median 1 (0–9) for CW; *p* = 0.496. Specifically, there was a 95% chance that the median number of cumulative Gd-enhancing lesions with the CW regimen was at most 1.2 times the median number of cumulative Gd-enhancing lesions with the FR schedule (Figure 2). The ratio of the median of the CW group on the median of the FR group and its relative two sided 90% CI was 1 (0.29; 1.07), allowing to significantly reject the null hypothesis of a ratio ≥1.2, and to confirm the non-inferiority of the CW regimen. Data related to the secondary endpoints of the first 9-month treatment period are summarized in Table 2. All secondary endpoints were met, although the study was not powered for any of them. The relapse RR, adjusted for baseline covariates, was comparable between CW and FR group (RR = 2.15, 95% CI: 0.64–7.18). Likewise, the mean number of cumulative new and enlarging T2-weighted lesions, the number of patients with at least an active scans, the number and volume of black holes and the median values of EDSS score did not show significant differences between the two study arms (respectively, *p* = 0.820; *p* = 0.865; *p* = 0.666; *p* = 0.626; and *p* = 0.201). The proportion of NAB + patients was similar between the two study arms: 8 (36%) in FR group and 5 (20%) in CW; *p* = 0.33 (data not shown).

**Table 2:** Secondary end points after 9 months of treatment.

**Outcomes**	**FR (*n* = 29)**	**CW (*n* = 27)**	***P*-value**
**CUMULATIVE NUMBER OF GD-ENHANCING LESIONS AT MRI**			
Mean ± SD	9.52 ± 17.01	5.85 ± 9.42	0.496^c^
Median (IQR)	3 (0–10)	1 (0–9)	
**CUMULATIVE NUMBER OF NEW AND ENLARGING T2-WEIGHTED LESIONS AT MRI**			
Mean ± SD	3.86 ± 6.76	3.48 ± 5.34	0.820^c^
Median (IQR)	1 (0–2)	2 (0–4)	
**NUMBER OF PATIENTS WITH AT LEAST AN ACTIVE SCAN**			
*N*	22	21	0.865^b^
			**RR (95%CI)^a^**
**RELAPSES**			
Mean ± SD	0.34 ± 0.66	0.52 ± 0.89	2.15 (0.64; 7.18)
Median (IQR)	0 (0–0)	0 (0–1)	
Range	0–2	0–3	
			***P*****-value^b^**
**EDSS SCORE**			
Mean ± SD	1.76 ± 1.33	1.33 ± 0.72	0.201
Median (IQR)	1.5 (1–1.78)	1 (1–1.56)	
Range	0–6.5	0–3	
	**(*****n*** **=** **24)**^**c**^	**(*****n*** **=** **25)**^**c**^	
**BLACK HOLES–NUMBER^d^**			
Mean ± SD	2.71 ± 4.56	1.24 ± 1.61	0.666
Median (IQR)	0 (0–4)	1 (0–2)	
Range	0–18	0–5	
**Black HOLES–OVERALL VOLUME (cm**^**3**^**)^d^**			
Mean ± SD	204.48 ± 396.98	489.11 ± 1488.12	0.626
Median (IQR)	0 (0–199.08)	0 (0–441.07)	
Range	0–1380.44	0–7357.59	

a *Relapse risk Ratio (RR) were obtained from Poisson model adjusting for the baseline variables (gender, age, disease duration, number of relapses before study entry, EDSS score, MRI metrics). After adjusting for covariates we analyzed 49 subjects with complete information*.

b *Non-parametric Wilcoxon rank-sum test (for variables with non-normal distribution)*.

c *The baseline demographic and clinical characteristics of this subgroup were not different from those of the 7 patients lost for this analysis*.

d *At month 12 of the study*.

The open-label extension phase lasted between 8 and 10 years, depending upon individual patient recruitment date. In this phase, all patient who remained on IFN-b 1b took the FR, while others shifted to the DMT that their neurologist deemed indicated. The shifting to a second-line treatment occurred in a minority of cases (7/27 in both groups). Results of the open-label phase, summarized in Table 3, showed no difference between CW and FR study arms in the median of annual relapses rate (*p* = 0.276), the median of EDSS score (*p* = 0.691), and the proportion of patients shifting to a progressive phase of disease (*p* = 0.715).

**Table 3:** Outcomes measures of the open label phase.

**Outcomes**	**FR (*n* = 27)**	**CW (*n* = 27)**	***p*-value**
**ANNUAL RELAPSE RATE**			
Mean ± SD	0.20 ± 0.21	0.40 ± 0.73	0.276^a^
Median (IQR)	0.14 (0–0.37)	0.25 (0–0.50)	
Range	0–0.7	0–3.5	
**EDSS SCORE**			
Mean ± SD	2.42 ± 2.14	2.40 ± 2.29	0.691^a^
Median (IQR)	1.5 (1–3.5)	1.5 (1–3.5)	
Range	0–7	0–7	
**SHIFTING TO A PROGRESSIVE DISEASE**			
*N* (%)	4 (0.14)	5 (0.18)	0.715^b^

a *Non-parametric Wilcoxon rank-sum test (for variables with non-normal distribution)*.

b *Chi-square test for categorical variables*.

No major adverse event was recorded throughout the trial. During the single-blind phase and the open-label extension of the trial the frequency and the nature of adverse events were within the established profile of IFN-b 1b, or the DMT that the patients shifted to, without differences between CW and FR study arms.

## Discussion

Our pilot work compared two IFN-b 1b regimens in RR-MS and showed non-inferiority of a calendar with therapeutic holiday with respect to the approved schedule. The outcome measures proved to be comparable between the two study arms for both the clinical-MRI end-points, and the development of NAB to IFN-b during the blind phase of the trial.

The clinical metrics gathered over the open-label phase lasting up to 10 years (during which most of patients remained under continuous IFN-b therapy, or were shifted to the DMT that their neurologist in charge considered indicated) were also comparable between the two groups, suggesting that beginning IFN therapy with a cyclic dose variation should not affect the long-term clinical progression of MS. Our finding points to the possibility of an IFN-b schedule with better therapeutic index and cost-effectiveness ratio, at least in cases with relatively benign disease, taking into account that there are ~30% of patients stable on IFN-b long-term treatment. The estimated saving for a year of the therapy with 1-month holiday every quarter (as in our trial design) is about 5,000 euros/patient (or 20,000 dollar/patient in US). These improvements also open new perspectives for the design of combination therapies, a much-needed step in a disease with multiple mechanisms of damage, and a timely advancement now that treatments directed against the neurodegenerative component of the disease are emerging (19). This perspective contributes to the current relevance of our study, although the present landscape of the first-line DMT, that include oral drugs, has actually restricted the indications of injectable DMT. In fact, interferons remain a relevant option within available treatments, as witnessed also by the development of new IFN-b formulations aimed at reducing the frequency of the injections (20). In this context, if therapeutic holidays should prove feasible also with this class of IFNs, administered once every 2 weeks, the convenience of these injectable therapies would be further increased.

Previous work suggested an increased efficacy of IFN-b at higher dose in contrasting disease activity (21–23). Moreover, in a daily clinical setting, a switch to higher dose of IFN-b may be in principle prescribed to obtain a better control of disease course, though with uncertain outcomes on the risk of further relapses or increased disability (24). In accord with a substantial amount of pre-clinical and clinical research in conditions other than MS (25), we now suggest that the therapeutic holidays sustain the biological impact of IFN-b over time (in spite of the lower overall dose) by periodically interrupting feedback mechanisms that are anticipated in chronic treatment regimens (the homeostatic response during chronic treatments is well-documented also at the clinical level by the reduction of the flu-like syndrome after the beginning of IFN-b therapies, of gastrointestinal symptoms and flushing during dimethyl fumarate treatment and of other side-effects with various other drugs). A study on the continuous vs. intermittent therapy for chronic hepatitis C with interferon alfa-2a supports this interpretation (26). Notably, the block of the homeostatic response was not as strong as to imply a clinically significant resurgence of the flu-like syndrome at the beginning of each new cycle of treatment. In fact, though we did not plan formal questionnaires to compare the quality of life between the two study arms, and we had no adherence problems in FR group, virtually all participants experiencing the CW regimen reported satisfaction for the regular withdrawal. Overall, these results are in line with expectations based on our transcriptomics study in PBMC from IFN-treated patients, that prompted this pilot trial. It showed both homeostatic responses to IFN-b administration (that we used to design the dynamics of drug holiday), and sustained and lasting effects on up- or down-regulated genes, that antagonize some pathogenic loops of MS (6). Previous work on drug holiday for other DMT in MS are rare, with contrasting or disappointing results (27–29); however, this trial suggests to deepen temporal dynamics underpinning their biological effects in MS course, with the aim of reducing the treatment burden or favoring combination therapies.

Concerning the potential limitations of this trial, we chose to leave patients under CW regimen on a “true” therapeutic holiday: they were not given placebo during the month of IFN withdrawal, thus remaining un-blinded on their treatment schedule. In fact, blindness of neuroradiologists and the assessing neurologist was deemed sufficient considering the study end points. On the other hand, a sort of nocebo effect due to expectation of lower treatment response to the CW regimen did not produce measurable changes, being met the end points of non-inferiority. Another point of caution is the small sample size of the trial; it allowed us to obtain statistical significance, but warns against definitive conclusions and may require confirmative studies. This is in line with the pilot nature of our study, that was designed to minimize the possibility that loss of efficacy might occur in a larger population under CW regimen. The open-label phase of the study has the expected limitations of a trial long-term extension: non-prospective design, possibly selective dropout rate, un-blinded assessment and poor monitoring, underpowered sample size. The loss of only 2 patients in FR group at long-term follow-up, and the fact that the shifting to a second-line treatment occurred in a minority of cases in both study arms indicate the validity of the long follow-up we chose. Also, the analysis of the categorical results of the open-label part of the study, that were adjusted for the baseline characteristics of participants, might help mitigate, at least in part, the above potential shortcomings. However, the data coming from the unblinded trial phase should be interpreted with caution.

Randomized, non-inferiority trials are rare in MS likely because of the challenges that these studies raise, as recently elucidated (30). Though exploratory, this trial may be relevant in the MS therapeutic field, providing information that might improve the schedule of IFN-beta administration in persons with RR-MS. This information is relevant for clinical practice (31) and for the design of poly-therapies [an approach that is beginning to be tested: reference (32), and ClinicalTrials.gov- NCT02907177] aimed at combating the various mechanisms of damage that compose the complex pathophysiology of MS.

## Data Availability

All datasets generated for this study are included in the manuscript and/or the supplementary files.

## Ethics Statement

The study was registered at the ClinicalTrials.gov (NCT00270816). The trial was conducted according to Good Clinical Practice guidelines and the Declaration of Helsinki. The protocol was approved by the local ethics committees and each patient provided written informed consent.

## Author Contributions

GR and MS were the study principal investigators. MS, GR, FB, CP, and SR conceived and designed the study. SR, MFe, MB, AF, VN, MM, and MFr were assessing neurologists. FB provided image analyses. RM, MC, and AB acquired and analyzed data on antibodies anti-IFN. All the authors were involved in the assessment and interpretation of the data. MFe, FM, and NV performed the statistical analyses. GR, SR, MFe, FB, FM, NV, CP, and MS contributed to write the manuscript.

### Conflict of Interest Statement

SR receives research support from Italian Multiple Sclerosis Foundation. FB serves in the Scientific Advisory Board of Merck-Serono. RM receives research support from Italian Multiple Sclerosis Foundation. MC received speaker honoraria from Biogen Idec, Merck, and Teva. MB received onorary fees from Teva, Sanofy, Biogen Idec, Merck, Novartis. AF received onorary fees from Teva and Novartis. VN received speaker honoraria from Teva, Biogen Idec, Bayer Schering, Merck, Almirall, Genzyme, Novartis. MM received honoraria for speaking, advisory board/consulting from Teva, Almirall, Biogen, Bayer Schering, Merck, Novartis Pharmaceuticals, Ultragenix, and financial support for research activities from Merck, Almirall, Teva and Novartis Pharmaceuticals. AB served on the scientific advisory boards of Almirall, Bayer, Biogen Idec, and Genzyme; received speaker honoraria from Biogen Idec, Genzyme, Novartis, Sanofi-Aventis, and Teva; his institution has received grant support from Bayer, Biogen Idec, Merck, Novartis, Teva, the Italian Multiple Sclerosis Society, Fondazione Ricerca Biomedica ONLUS, and San Luigi ONLUS; is on the editorial board of Multiple Sclerosis International, Progress in Neuroscience, Dataset Papers in Neuroscience, Journal of Multiple Sclerosis, Neurology, and Therapy, and Multiple Sclerosis and Demyelinating Disorders; and received research support from Regione Piemonte, Italian Multiple Sclerosis Society, Associazione Ricerca Biomedica ONLUS, and San Luigi ONLUS. CP has received consulting and/or lecture fees and/or research funding and travel grant from Almirall, Bayer Schering, Biogen Idec, Genzyme, Merck Serono, Novartis, Roche, and Teva. MS receives research support from Italian Multiple Sclerosis Foundation (FISM) and research support with honorary fees from Teva, Bayer Schering, Genzyme, Biogen Idec, Merck and Novartis. GR receives research support from Italian Multiple Sclerosis Foundation (FISM). The remaining authors declare that the research was conducted in the absence of any commercial or financial relationships that could be construed as a potential conflict of interest.

## Abbreviations

ARR: annualized relapses rate
BH: black holes
cBHs: chronic black holes
CDMS: clinically definite multiple sclerosis
CELs: contrast enhancing lesions
CI: Confidence Interval
CPE: cytopathic effect
CW: cyclic withdrawal
D-MEM, Dulbecco's modified eagle's medium DMT: disease modifying therapies
EDSS, Expanded Disability Status Score. CW: cyclic withdrawal
EMC: encephalomyocarditis virus
FCS, fetal calf serum. FLAIR, fast fluid- attenuated inversion-recovery FR: full regimen
HR: Hazard Ratio
Gd: gadolinium
IFN-b: interferon beta
IQR: Interquartile Range
LOCF: last observation carried forward
MRI: magnetic resonance imaging
MS, Multiple sclerosis NAB: Neutralizing antibodies
PBMC: peripheral blood mononuclear cells
RR: risk ratio
RR-MS: relapsing remitting multiple sclerosis
SE: spin-echo
T1W: T1-weighted
T2W: T2-weighted
TRU: 10-fold reduction unit.
